# Inflammatory tropism in COVID-19: a comparative analysis of Delta and Omicron variants

**DOI:** 10.1186/s12865-025-00772-x

**Published:** 2025-11-14

**Authors:** Afshin Samiei, Ali Jandaghi, Mehdi Hassani Azad, Mahmood Khayatian, Narges Khaghanzadeh

**Affiliations:** 1 Endocrinology and Metabolism Research Center, Hormozgan University of Medical Sciences, Bandar Abbas, Iran; 2 Department of Immunology, Faculty of Medicine, Hormozgan University of Medical Sciences, Bandar Abbas, Iran; 3 Student Research Committee, Hormozgan University of Medical Sciences, Bandar Abbas, Iran; 4 Infectious and Tropical Diseases Research Center, Hormozgan Health Institute, Hormozgan University of Medical Sciences, Bandar Abbas, Iran; 5 Molecular Medicine Research Center, Hormozgan Health Institute, Hormozgan University of Medical Sciences, Bandar Abbas, Iran

**Keywords:** COVID-19, Delta, Omicron, Anti-MPO, Anti-PR3, Anti-GBM, NLR, ANCA

## Abstract

**Background:**

The clinical presentation of COVID-19 varies significantly by viral variant; the Delta variant often causes severe lung inflammation, whereas Omicron tends to result in less severe respiratory disease but may more readily affect other organs. The autoimmune mechanisms behind these variant-specific complications, particularly the potential role of anti-neutrophil cytoplasmic (ANCA) antibodies, are still not well defined. This study investigated myeloperoxidase (MPO), proteinase 3 (PR3), and glomerular basement membrane (GBM) antibodies in patients infected with the Delta and Omicron SARS-CoV-2 variants to evaluate their associations with specific clinical complications, such as renal or respiratory involvement.

**Methods:**

Samples were collected during Delta and Omicron outbreaks from hospitalized COVID-19 patients (40 Delta, 40 Omicron) and 40 healthy controls. Serum autoantibodies (MPO, PR3, GBM) were measured via ELISA, and laboratory/clinical data were collected.

**Results:**

All autoantibody levels remained within normal limits. Despite statistical significance (*P* < 0.05), effect sizes were small (η²=0.085–0.180). Anti-MPO was highest in the Delta group (1.08 U/mL) vs. Control (0.71 U/mL) and Omicron (0.77 U/mL). Anti-PR3 was lowest in the Delta group (0.83 U/mL) vs. Control (1.52 U/mL). Anti-GBM was lowest in the Omicron group (1.48 U/mL) vs. Control (3.48 U/mL) and Delta (3.10 U/mL).

Clinically, the Delta group exhibited significantly lower oxygen saturation (92.28% ± 8.69) than the Omicron group (96.15% ± 2.77; *P* = 0.009). Markers of inflammation and tissue damage—including C-reactive protein (CRP), lactate dehydrogenase (LDH), aspartate aminotransferase (AST) and alanine aminotransferase (ALT), and coagulation parameters—were markedly elevated in the Delta group compared to the Omicron group (*P* < 0.001). Conversely, creatinine was significantly higher in the Omicron group (*P* < 0.001).

**Conclusion:**

While autoantibody levels remained normal, clinical profiles diverged significantly between variants. The Delta variant was associated with heightened systemic inflammation, whereas Omicron was associated with greater organ involvement, suggesting the possibility of distinct pathogenic mechanisms.

**Supplementary Information:**

The online version contains supplementary material available at 10.1186/s12865-025-00772-x.

## Introduction

The COVID-19 pandemic has significantly impacted patient care, particularly for individuals with immune suppression, who are more susceptible to infection. Cases of ANCA-associated vasculitis (AAV) during acute COVID-19 have been reported [1, 2], raising concerns about the unclear impact of COVID-19 on AAV patients, especially since AAV can mimic COVID-19 symptoms [1, 3–5]. Investigating ANCA autoantibodies, commonly classified into two main types myeloperoxidase (MPO) and proteinase 3 (PR3), is crucial as they correlate with organ involvement [6]. Additionally, anti-glomerular basement membrane (GBM) antibodies are present in a subset of ANCA-positive patients, with 5–9% of AAV patients showing detectable circulating GBM antibodies [7, 8]. Existing evidence highlights the association between autoantibodies and COVID-19, emphasizing their clinical and diagnostic relevance in predicting organ involvement, particularly in the context of long COVID [1, 9, 10]. Recent evidence links COVID-19 to the onset of autoimmune diseases, including anti-PR3 and anti-MPO vasculitis in pediatric cases. For example, anti-PR3 has been associated with acute vasculitic events, such as a case of severe colonic damage in an asymptomatic teenager, although a pre-existing subclinical condition could not be ruled out [11]. Several case reports have documented anti-MPO vasculitis with pulmonary and renal involvement in pediatric patients who tested positive for COVID-19 [3, 12]. Furthermore, cases of anti-GBM vasculitis have emerged post-vaccination, highlighting the complex interactions between infections, autoimmune responses, and vaccination [12–14]. This study aimed to compare the levels of anti-PR3, anti-MPO, and anti-GBM autoantibodies in patients hospitalized with the Omicron variant of COVID-19 against those with the Delta variant, and to examine their correlations with clinical symptoms and laboratory parameters.

## Materials and methods

### Patients

Serum samples were collected from COVID-19 ICU patients at Shahid Mohammadi Hospital, Iran, during Delta (*n* = 40; Winter 2020–Summer 2021) and Omicron (*n* = 40; Spring–Summer 2022) waves, per WHO/Iranian Health Ministry reports. Controls (*n* = 40) were asymptomatic, PCR-negative individuals selected from occupational health screenings, with SpO₂ values within the normal range (95–100%).

Hospitalized COVID-19 patients with fever (≥ 37.8 °C) and at least one respiratory symptom (respiratory rate > 24/min, SpO₂ <94%, or PaO₂/FiO₂ <300 mmHg) were included. All cases were PCR-confirmed with CT evidence of lung involvement. Patients with autoimmune diseases, skin disorders, or negative PCR results were excluded. The study was approved by the ethics committee of Hormozgan University of Medical Sciences in accordance with the Declaration of Helsinki (approval code: IR.HUMS.REC.1400.376). Informed consent was obtained from all participants.

### Data collection and definitions

All clinical data, including underlying conditions, medications, COVID-19 vaccination status, symptoms, and organ involvement, were extracted from hospital medical records. Clinical and laboratory parameters were recorded at admission for all study groups (Control, Delta, and Omicron). Organ involvement was defined as clinically significant dysfunction in major organ systems, confirmed through laboratory testing, imaging studies, and/or clinical symptoms based on standardized hospital protocols.

### Sample collection and processing

For autoantibody quantification, 10 mL venous blood samples were collected. Serum samples were obtained, aliquoted to minimize freeze-thaw cycles, and stored at −70 °C for approximately 60 days prior to analysis for each group. The concentrations of serum autoantibodies (MPO, PR3, GBM) were quantified using ELISA kits: AESKULISA PR3-sensitive-c-ANCA (REF: 3302), AESKULISA MPO-p-ANCA (REF: 3303), and for Anti-GBM antibodies were quantified using ORGANTEC’s ELISA kit (REF: ORG 250 − 24) on an Alegria automated platform. (Negative: <20 U/mL for Anti-GBM; <5 U/mL for Anti-MPO/PR3).

### Statistical analysis

Data were analyzed using SPSS v.22 (IBM Corp.). Continuous variables were assessed for normality using Kolmogorov-Smirnov and Shapiro-Wilk tests. Normally distributed variables are presented as mean ± standard deviation (SD) and analyzed using parametric tests (ANOVA with post-hoc Tukey tests for multiple comparisons, MANOVA for multivariate analysis). Non-normally distributed variables are reported as median (interquartile range) and analyzed using non-parametric tests (Kruskal-Wallis with Dunn’s post-hoc tests). Categorical variables were compared using χ² (chi-square) tests or Fisher’s exact test as appropriate. Significance was defined as *P* < 0.05.

## Results

### Demographic findings

The three groups did not show significant differences in age (57.68 ± 17.76), gender (60 females vs. 60 males; *P* = 0.97) or smoking status (Supplementary Table 1). Table 1 presents the demographic findings and other descriptive data of the patients. As illustrated in Table 1, the prescription of corticosteroids, antivirals, and anticoagulants was significantly higher in the Delta group. Disease symptoms like dyspnea and lung involvement were significantly more prevalent in the Delta group compared to the Omicron group. Conversely, organ involvement and sore throat were significantly more common in the Omicron group than in the Delta group (Table 1).

**Table 1:** Comparative analysis of Comorbidities, Treatments, and Clinical outcomes between Delta and Omicron COVID-19 cases

Variable	Delta (*n* = 40)	Omicron (*n* = 40)	χ²	*P*-value	Effect Size (φ/V)
Comorbidities					
Anemia	2.5%	10.3%	2.004	0.157	0.159
Diabetes Mellitus	15.0%	22.5%	0.738	0.390	0.096
Hypertension	30.0%	37.5%	0.503	0.478	0.079
Medications					
Corticosteroid Use	85.0%	65.0%	4.267	0.039*	0.231
Antiviral Use	95.0%	72.5%	7.440	0.006**	0.305
Anticoagulant Use	100.0%	80.0%	8.889	0.003**	0.333
Symptoms					
Dyspnea	65.0%	37.5%	6.054	0.014*	0.275
Pharyngeal Discomfort	0.0%	20.0%	8.889	0.003**	0.333
Clinical Findings					
Organ Involvement	5.0%	22.5%	5.165	0.023*	0.254
Lung Involvement	92.5%	75.0%	4.501	0.034*	0.237

*P < 0.05, ** *P* < 0.01. Data presented as percentages within variant groups. χ² tests (Pearson) were used for comparisons unless noted. *Fisher’s exact test was applied when > 20% of cells had expected counts < 5. Missing data ranged from 0–34.2.2% across variables (complete case analysis used). Effect sizes reported as Phi (φ) for 2 × 2 tables and Cramer’s V for larger contingency tables

The comparison of all other baseline clinical characteristics revealed no significant differences between the groups. For full details, see Supplementary Table 1.

Vaccination dosage differed significantly between groups (*P* < 0.001). The Delta group was entirely unvaccinated (median = 0, IQR = 0–0), whereas both the Control and Omicron groups had a median of 3 doses (IQR = 2–3), with no difference between them (*P* = 1.000) (Supplementary Table 1).

### Laboratory findings

Table 2 compares the laboratory parameters and autoantibody levels among the three groups. According to the Table 2, CRP levels and liver enzymes including AST and ALT were significantly higher in the Delta group compared to the other two groups (*P* < 0.001). Conversely, creatinine (*P* < 0.001) was significantly elevated in the Omicron group compared to the others. Additionally, lactate dehydrogenase (LDH) levels were significantly (*P* < 0.001) higher in the Delta group than that in the Omicron group. Furthermore, markers of coagulation dysregulation, including prothrombin time (PT), partial thromboplastin time (PTT), and international normalized ratio (INR), were all significantly elevated in the Delta group compared to the Omicron group (*P* < 0.05).

**Table 2:** Comparison of Hematological, Biochemical, and Immunological parameters among Control, Delta, and Omicron groups

Parameter	Control (*n* = 40)	Delta (*n* = 40)	Omicron (*n* = 40)	*P*-value
Inflammatory Markers				
WBC (×10⁹/L)	7.54 ± 1.64	7.04 ± 3.27	7.30 ± 3.54	0.752
Neutrophils (%)	49.99 ± 5.96	72.09 ± 14.34	72.62 ± 14.70	< 0.001
Lymphocytes (%)	42.40 ± 6.25	20.84 ± 12.01	20.39 ± 11.88	< 0.001
NLR	1.22 ± 0.32	5.06 ± 3.48	5.37 ± 3.76	< 0.001
CRP (mg/L)^a^	5.00 (3.50–7.00)	46.56 (29.50–65.30)	32.00 (13.40–46.60)	< 0.001
ESR (mm/hr)^a^	10.00 (5.00–15.00)	49.50 (35.00–65.00)	39.00 (25.00–52.00)	< 0.001
Ferritin (µg/L)^a^	-	476.26 (315.50–650.00)	111.26 (70.50–180.00)	0.003
Liver Function				
LDH (U/L)^a^	-	650.00 (530.00–850.00)	337.25 (280.00–410.00)	< 0.001
AST (U/L)^a^	22.25 (18.00–27.00)	42.75 (32.00–55.00)	29.50 (23.00–37.00)	< 0.001
ALT (U/L)^a^	16.00 (12.00–22.00)	41.75 (30.00–55.00)	29.00 (20.00–38.00)	< 0.001
Coagulation Profile				
D-dimer (ng/mL)^a^	-	232.4 [92.3, 691.8]	135.6 [72.6, 315.5]	0.085
PT (sec)	-	14.03 ± 3.24	12.75 ± 1.07	0.020
PTT (sec)	-	40.67 ± 17.44	33.32 ± 8.00	0.018
INR	-	1.24 ± 0.51	1.03 ± 0.15	0.016
Renal Function				
BUN (mg/dL)^a^	24.25 (19.00–32.00)	32.70 (25.00–40.00)	40.00 (32.00–48.00)	0.157
Creatinine (mg/dL)^a^	0.80 (0.70–0.90)	0.90 (0.80–1.10)	1.20 (1.00–1.50)	< 0.001
Autoantibodies				
Anti-GBM (IU/mL)	3.48 ± 2.00	3.10 ± 1.50	1.48 ± 1.92	< 0.001
Anti-PR3 (IU/mL)	1.52 ± 0.55	0.83 ± 0.43	1.04 ± 0.21	< 0.001
Anti-MPO (IU/mL)	0.70 ± 0.14	1.08 ± 0.27	0.77 ± 0.16	< 0.001

Data presented as Mean ± Standard Deviation/^a^ Median (Interquartile Range). *P*-values were derived from [One-Way ANOVA or Kruskal-Wallis Test]. *NLR* Neutrophil-to-Lymphocyte Ratio, *LDH* Lactate Dehydrogenase, *AST* Aspartate Aminotransferase, *ALT* Alanine Aminotransferase, *PT* Prothrombin Time, *PTT* Partial Thromboplastin Time, *INR* International Normalized Ratio, *BUN* Blood Urea Nitrogen, *GBM* Glomerular Basement Membrane, *PR3* Proteinase 3, *MPO* Myeloperoxidase

A dash (–) indicates that data for this parameter was not collected or is not applicable for the Control group

The neutrophil-to-lymphocyte ratio (NLR) was also assessed across the groups (with an NLR above 3 indicating inflammation or disease) [15]. Although mean NLR was numerically higher in the Omicron group (5.37) than the Delta group (5.06), this difference was not statistically significant (*P* = 0.886). Importantly, both variant groups showed significantly elevated NLR versus controls (*P* < 0.001), supporting NLR’s value in distinguishing COVID-19 infection from non-infection rather than differentiating between these two variants.

Despite statistically significant inter-group differences, mean serum levels of anti-MPO, anti-PR3, and anti-GBM autoantibodies for all groups remained within established normal reference ranges, indicating a lack of clinical elevation. Anti-PR3 levels were significantly lower in the Delta (0.83 ± 0.43 IU/mL) and Omicron (1.04 ± 0.21 IU/mL) groups compared to Controls (1.52 ± 0.55 IU/mL) (*P* < 0.001), though the Delta-Omicron difference was not significant (*P* = 0.06) likely due to high Delta variability. Anti-MPO levels were highest in the Delta group (1.08 ± 0.27 IU/mL) compared to both Control (0.70 ± 0.14 IU/mL) and Omicron (0.77 ± 0.16 IU/mL) groups (*P* < 0.001). Anti-GBM levels were significantly lower in the Omicron group (1.48 ± 1.92 IU/mL) than in both the Delta (3.10 ± 1.50 IU/mL) and Control (3.48 ± 2.00 IU/mL) groups (*P* < 0.001). The small effect sizes (η² = 0.085–0.180) associated with these differences suggest they represent natural variation in constitutive production rather than a clinically meaningful effect.

### Vital signs

As shown in Table 3, vital signs analysis revealed comparable heart rate and blood pressure between Delta and Omicron groups. The Delta group exhibited significantly lower oxygen saturation (95.0 (90.8–96.8)) than the Omicron group (97.0 (96.0–98.0); *P* < 0.001). The control group exhibited oxygen saturation (SpO₂) values within the normal physiological range (95–100%).

**Table 3:** Comparative analysis of vital parameters between delta and Omicron groups

Parameter	Group	*N*	Mean ± SD/Median (IQR)*	Test Statistic	*P*-value
Heart Rate (bpm)	Delta	40	84.78 ± 11.53	t = −0.030	0.976
	Omicron	40	84.85 ± 11.16	df = 78	
Respiratory Rate (bpm)	Delta	40	18.75 (17.50–20.00)*	U = 548.5	0.012*
	Omicron	40	18.65 (16.00–21.25.00.25)*	Z = −2.505	
Oxygen Saturation (%)	Delta	40	95.0 (90.8–96.8)*	U = 469.5	0.001*
	Omicron	40	97.0 (96.0–98.0)*	Z = −3.212	
Systolic BP (mmHg)	Delta	40	120.0 (110.0–130.0)*	U = 778.5	0.911
	Omicron	40	120.0 (110.0–130.0) *	Z= −0.112	
Diastolic BP (mmHg)	Delta	40	79.0 (70.0–80.0) *	U = 698.5	0.309
	Omicron	40	80.0 (70.0–80.0) *	Z = −1.017	

Data are presented as Mean ± SD/Median (IQR)*. Normally distributed data: Mean ± SD with independent t-test results. Non-normal data: Median (IQR) with Mann-Whitney U test results (U and Z statistics). The significance level is set at *P* < 0.05. bpm (beats per minute), mmHg (millimeters of mercury)

### MANOVA analysis

Multivariate analysis results (Table 4) demonstrated significant associations between various autoantibody levels and inflammation markers. The corrected model revealed strong associations between inflammatory markers and autoantibody production. NLR > 3 demonstrated the most robust effect (F = 8.391, *P* < 0.001, η²=0.778), explaining 77.8% of variance in inflammation. Both Anti-GBM (F = 2.282, *P* = 0.006, η²=0.488) and Anti-MPO (F = 2.488, *P* = 0.003, η²=0.510) showed significant moderate-to-large effects. Neutrophil percentage emerged as a key driver, predicting both NLR > 3 (F = 6.138, *P* = 0.016) and Anti-MPO levels (F = 9.980, *P* = 0.003). Although intercept terms for baseline autoantibodies were statistically significant (*P* < 0.05), their small effect sizes (η²=0.085–0.180) suggested limited biological relevance compared to clinical predictors. Notably, prolonged PTT associated with Anti-MPO (F = 4.492, *P* = 0.039), indicating potential coagulation-autoimmunity interactions.

**Table 4:** Multivariate analysis of variance (MANOVA) of inflammatory and ANCA antibodies

Source	Dependent Variable	F	*P*-value	Partial η²	Interpretation
Corrected Model	NLR > 3 (Inflammation)	8.391	< 0.001	0.778	Very strong inflammatory response, explaining 77.8% of variance
	Anti-GBM (U/mL)	2.282	0.006	0.488	Strong association with anti-GBM autoantibody production
	Anti-MPO (U/mL)	2.488	0.003	0.510	Strong association with anti-MPO autoantibody production
Intercept	Anti-GBM (U/mL)	5.096	0.028	0.085	Baseline anti-GBM levels present but clinically minor effect
	Anti-MPO (U/mL)	12.109	0.001	0.180	Significant baseline anti-MPO levels with small-medium effect
Neutrophil (%)	NLR > 3 (Inflammation)	6.138	0.016	0.100	Neutrophils contribute significantly to inflammatory status with small- medium effect
	Anti-MPO (U/mL)	9.980	0.003	0.154	Neutrophil percentage predicts anti-MPO levels with medium effect
PTT (s)	Anti-MPO (U/mL)	4.492	0.039	0.076	Coagulation disturbance weakly associated with anti-MPO

Source: Factors or variables analyzed, F: F-statistic for the overall model or predictor, Partial η²: Effect size (values closer to 1 indicate a stronger effect). Significance level (*P* < 0.05 indicates statistical significance). Intercept significance reflects non-zero baseline autoantibody production, but clinical predictors account for 5–10× more variance (see Partial η²)

## Discussion

In the present study, we investigated the presence of autoantibodies across two variants of COVID-19, specifically focusing on the Delta and Omicron variants. The study elucidates that although autoantibody (anti-MPO) levels remained within normal range, their significant association with neutrophilic inflammation (NLR) and coagulation (PTT) suggests a role in COVID-19 thromboinflammation. The timing of sampling early in hospitalization and widespread corticosteroid use likely suppressed titers, masking their full clinical relevance. This underscores that even sub-clinical autoantibody responses may contribute to the immunopathology of SARS-CoV-2, particularly in severe variants. This observation aligns with previous studies indicating that ANCA antibody levels can serve as indicators of underlying inflammatory processes that may contribute to organ involvement, such as renal impairment and pulmonary complications [8, 12, 16–18]. Furthermore, previous reports confirm that corticosteroids suppress autoantibody levels [2, 3, 11–14, 19, 20]. Collectively, this suggests that while autoantibody production is a significant phenomenon, a single measurement of their concentration may not fully capture their pathogenic potential.

Multivariate analysis revealed significant relationships between anti-MPO antibodies and inflammatory markers. Although baseline autoantibody levels were statistically significant, their small effect sizes (η² = 0.085–0.180) suggest these primarily reflect constitutive production rather than clinically meaningful variation. This aligns with studies reporting low-titer autoantibodies in COVID-19 patients, including vaccinated individuals, though their pathophysiological role remains unclear when effect sizes fall below typical clinical relevance thresholds [21–23]. Stronger associations were observed with active clinical predictors (e.g., NLR, neutrophil %), which likely better reflect disease-associated autoimmunity.

These findings must be interpreted within the clinical context of the Delta variant’s more severe phenotype, characterized by significantly higher PTT levels, alongside greater use of corticosteroids, antivirals, and anticoagulants compared to the Omicron group. This suggests that the observed associations are driven by a state of heightened thromboinflammation, particularly in Delta-infected patients. These results extend current understanding of ANCA-associated vasculitis (AAV) pathogenesis as described by Drynda et al. [24]. Neutrophils emerged as a central driver in our analysis. Neutrophil percentage significantly predicted both elevated NLR (>3) and anti-MPO levels, reinforcing the core role of neutrophils in MPO-ANCA vasculitis. Neutrophils are not only a source of MPO autoantigen but also active effectors of vascular damage [24]. An elevated neutrophil population was common to both variants compared to controls.

The Delta variant was associated with coagulopathy characterized by prolonged PTT and PT levels. This profile reflects a pronounced thromboinflammatory state. Correspondingly, the Delta group exhibited more pronounced anti-MPO levels (albeit within normal range), suggesting a pathophysiological link between neutrophil activation and the variant’s heightened thromboinflammatory response. Our MANOVA model further indicated an association between prolonged PTT and anti-MPO levels. Prolonged PTT has been previously associated with AAV in the literature [25]. These findings align with established models of thromboinflammation, such as in AAV, where neutrophil activation, NETosis, and tissue factor expression promote thrombin generation, platelet activation, and inflammatory amplification [24, 26]. Furthermore, PTT is significantly elevated in the Delta group compared to the Omicron group. These findings are consistent with the established relationships between inflammatory and coagulation parameters and disease severity, aligning with the results reported by Esmaeel H. et al., which indicated elevated levels of PTT in severely affected COVID-19 patients compared to those with non-severe cases [27].

This neutrophil-driven inflammation was marked by significantly elevated neutrophil counts and NLR in both variant groups. The association between high NLR and anti-MPO levels suggests that systemic neutrophilia is linked to autoantibody production, mirroring the relationship seen in ANCA-associated vasculitis and indicating a common pathway of neutrophil-derived immunopathology [28–30]. Consequently, the Delta group’s prolonged coagulation time, as evidenced by significantly higher PTT, was paralleled by its more pronounced NLR and anti-MPO levels.

Both the Omicron and Delta variants exhibited significantly elevated NLR compared to normal controls. MANOVA analysis indicates a 77.8% effect of this inflammatory marker in both variants. This finding further supports the association between NLR and increased inflammation in COVID-19 [31], highlighting its value as an indicator of systemic inflammation. The lack of a significant difference between the Delta and Omicron groups may suggest that both variants elicit a comparable inflammatory response, despite differences in clinical presentation.

Our results indicate that the Delta variant exhibited more pronounced levels of anti-MPO, compared to the other groups. This finding aligns with previous studies that report the involvement of autoantibodies in vasculitis; however, most of these studies are case reports that do not specify the types of COVID-19 variants involved, often only indicating the time frame of the study [1, 17, 20, 23].

Although anti-PR3 levels across all groups remained within the normal range, the significantly lower levels observed in the Delta group compared to Controls and the Omicron group are a notable finding. This attenuation is likely iatrogenic and could be a consequence of substantially higher corticosteroid use in Delta patients (85.0%) compared to the Omicron group (65.0%), a treatment known to suppress autoantibody production [4, 18, 32]. The higher anti-PR3 in controls, while statistically significant, is of uncertain clinical relevance as all mean values remained well within the manufacturer-defined negative range (< 5 U/mL).

Based on our findings and the emerging literature, while rare case reports link COVID-19 to new-onset anti-GBM disease [22], we did not detect clinically elevated anti-GBM levels in our groups. This suggests that while a causal relationship is biologically plausible, it remains a rare complication.

While the Delta variant exhibited higher levels of inflammatory markers such as CRP, AST, and ALT—indicating a potential for greater liver involvement—the Omicron variant showed significantly elevated levels of creatinine, which is marker for renal function. While both variants showed comparable rates of kidney pathology (Delta: 5% vs. Omicron: 10%; *P* = 0.396 (Suplementary Table 1)), the elevated creatinine in Omicron cases could potentially reflect subclinical injury or alternative mechanisms of renal involvement. This observation, coupled with the established link between COVID-19 and renal autoimmunity—where MPO-ANCA is frequently reported in pauci-immune glomerulonephritis cases [29]—highlights a complex immunopathological relationship. however, our study did not find elevated anti-GBM or ANCA levels beyond the normal clinical range.

The elevated levels of CRP and liver enzymes in the Delta group compared to the Omicron and Control groups suggest a more pronounced inflammatory response, which may correlate with the severity of the disease. Other studies have indicated that elevated levels of liver enzymes are associated with increased severity in COVID-19 patients [33]. Conversely, the higher levels of creatinine in the Omicron group warrant further investigation into potential renal implications, as these findings could reflect differing pathophysiological mechanisms between the variants. These results are supported by a previous study conducted in China, which demonstrated increased levels of creatinine in Omicron patients compared to those with the Delta variant [34]. Although blood urea nitrogen (BUN) was elevated in the Omicron group, the difference compared to the Delta group was not statistically significant.

The level of LDH in the Delta group is significantly higher than that in the Omicron group. The potential role of LDH as a biomarker for COVID-19 severity has been explored in several studies, revealing a strong association that supports our findings [27, 35].

A significant difference in oxygen saturation was observed, with the Omicron group demonstrating higher levels than the Delta group. This is consistent with reports of Omicron’s association with less severe pulmonary disease, including a higher incidence of unilateral lung involvement [36]. Although this pattern may contribute to better-preserved oxygenation, it does not preclude severe radiological disease in vulnerable populations. The greater hypoxemia in Delta patients likely reflects its more frequent and extensive lung involvement.

In contrast to the marked differences in respiratory status and inflammation, heart rate was nearly identical between the Delta and Omicron groups (*P* = 0.97), suggesting a variant-specific impact on pulmonary function rather than cardiovascular system.

While any tissue can be affected, the respiratory tract and kidneys are the most commonly involved in ANCA-associated vasculitis (AAV) [37–39]. Our study presents a notable discrepancy from previous reports, as we found that the Omicron variant was associated with higher organ involvement (22.5%, *P* = 0.023) compared to the Delta variant. A recent study investigating the clinical characteristics of the Omicron variant of SARS-CoV-2 analyzed medical charts from 384 Omicron-infected patients in China. While initial symptoms were similar between Omicron and Delta variants, the findings indicate that Omicron is associated with relatively lower organ involvement compared to Delta [34, 36].

The results from this study underscore the critical role of various biomarkers, particularly anti-MPO, in predicting inflammation and related conditions in COVID-19 patients. The significant differences observed in laboratory parameters such as CRP, liver enzymes (AST and ALT), and renal function marker (creatinine) highlight the distinct inflammatory profiles associated with the Delta and Omicron variants.

This study highlights the need for larger, variant-specific research to clarify the role of ANCA antibodies in COVID-19 organ involvement, particularly regarding long-term renal and hepatic involvement. Future research should consider investigating the levels of autoantibodies in COVID-19 patients who have not received corticosteroids or other anti-inflammatory treatments, as well as conducting studies on larger samples to achieve more accurate and generalizable results.

## Conclusion

This study highlights significant differences in inflammatory markers and autoantibody levels among COVID-19 patients infected with different variants, particularly the Delta and Omicron variants. Elevated levels of CRP and liver enzymes in the Delta group indicate a more pronounced inflammatory response, while increased creatinine levels in the Omicron group suggest renal involvement. The findings underscore the importance of integrating laboratory parameters and autoantibody levels to understand the inflammatory landscape of COVID-19, emphasizing the need for further research to explore the implications of these biomarkers on treatment strategies and patient outcomes. Ultimately, understanding these differences is crucial for tailoring clinical approaches and improving public health responses to future COVID-19 variants.

## Supplementary Information

Supplementary Material 1.

## Data Availability

The datasets used and/or analysed during the current study are available from the corresponding author on reasonable request.
