# Supplementary material for: Inflammatory tropism in COVID-19: a comparative analysis of Delta and Omicron variants
Source: BMC Immunol. 2025 Nov 14;26:92. doi: 10.1186/s12865-025-00772-x (PMC12619456; doi:10.1186/s12865-025-00772-x)
Supplement: Supplementary file 1 — Supplementary Material 1. [file 12865_2025_772_MOESM1_ESM.docx]

**Supplementary Table 1: Comparative Analysis of Comorbidities, Treatments, and Clinical Outcomes Between Groups**

| **Variable** | **Delta Group (n=40)** | **Omicron Group (n=40)** | **χ² (df)** | **P-value** |
| --- | --- | --- | --- | --- |
| **Smoking** | 5% (2) | 15% (6) | 2.22 (1) | 0.136 |
| **Comorbidities** |  |  |  |  |
| Hypothyroidism | 7.5% (3) | 15.0% (6) | 1.13 (1) | 0.288 |
| Asthma | 0.0% (0) | 5.0% (2) | 2.05 (1) | 0.152 |
| Cardiovascular Disease | 25.0% (10) | 22.5% (9) | 0.07 (1) | 0.793 |
| Neurologic Disorders | 7.5% (3) | 7.5% (3) | 0.00 (1) | 1.000 |
| Hyperlipidemia | 0.0% (0) | 5.0% (2) | 2.05 (1) | 0.152 |
| Tuberculosis | 0.0% (0) | 2.5% (1) | 1.01 (1) | 0.314 |
| Renal Disease | 5.0% (2) | 10.0% (4) | 0.72 (1) | 0.396 |
| **Medications** |  |  |  |  |
| Antilipid | 65.0% (26) | 62.5% (25) | 0.05 (1) | 0.816 |
| PPI/H2 Blocker | 87.5% (35) | 87.5% (35) | 0.00 (1) | 1.000 |
| Antihistamine | 22.5% (9) | 30.0% (12) | 0.58 (1) | 0.446 |
| Antibiotic | 60.0% (24) | 57.5% (23) | 0.05 (1) | 0.820 |
| Antiasthma | 45.0% (18) | 27.5% (11) | 2.65 (1) | 0.104 |
| Antihypertensive | 30.0% (12) | 30.0% (12) | 0.00 (1) | 1.000 |
| Renal Therapy | 5.0% (2) | 2.5% (1) | 0.35 (1) | 0.556 |
| Rheumatoid Treatment | 0.0% (0) | 5.0% (2) | 2.05 (1) | 0.152 |
| **Symptoms** |  |  |  |  |
| Fever | 57.5% (23) | 67.5% (27) | 0.85 (1) | 0.356 |
| Cough | 62.5% (25) | 62.5% (25) | 0.00 (1) | 1.000 |
| Sputum | 17.5% (7) | 32.5% (13) | 2.40 (1) | 0.121 |
| Bloody Sputum | 0.0% (0) | 2.5% (1) | 1.01 (1) | 0.314 |
| Chest Pain | 10.0% (4) | 10.0% (4) | 0.00 (1) | 1.000 |
| Myalgia | 75.0% (30) | 60.0% (24) | 2.05 (1) | 0.152 |
| Abdominal Pain | 7.5% (3) | 7.5% (3) | 0.00 (1) | 1.000 |
| Diarrhea | 20.0% (8) | 12.5% (5) | 0.83 (1) | 0.363 |
| Vomiting | 27.5% (11) | 32.5% (13) | 0.24 (1) | 0.626 |
| Dizziness/Headache | 32.5% (13) | 22.5% (9) | 1.00 (1) | 0.317 |
| Kidney Pathology | 5.0% (2) | 10.0% (4) | 0.72 (1) | 0.396 |
| Renal Replacement | 5.0% (2) | 7.5% (3) | 0.21 (1) | 0.644 |
| **Vaccine Dosage Comparison** | **n** | **Median** | **IQR** | **^*^ Post-hoc Comparisons (Mann-Whitney U)** |
| **Control** | 40 | 3.0 | 2.0-3.0 | vs. Delta: U = 0.000, **P < 0.001** |
| **Delta** | 40 | 0.0 | 0.0-0.0 | vs. Omicron: U = 0.000, **P < 0.001** |
| **Omicron** | 40 | 3.0 | 2.0-3.0 | vs. Control: U = 800.000, P = 1.000 |

**Data are presented as % (n) unless otherwise specified. χ² = Chi-square test; df = degrees of freedom. IQR = Interquartile Range. The ^*^ Bonferroni correction adjusted the significance level for post-hoc tests to P< .0167.**
